# Mean Distance of Mental Foramen from Inferior Border of Mandible among Patients Visiting the Outpatient Dental Department in a Tertiary Care Centre

**DOI:** 10.31729/jnma.8289

**Published:** 2023-10-31

**Authors:** Preeti Singh, Biplob Adhikari, Sushmit Koju, Sujaya Gupta, Megha Pradhan, Deepa Gurung, Ujjwal Joshi

**Affiliations:** 1 Department of Oral Pathology and Microbiology, Kathmandu Medical College and Teaching Hospital, Duwakot, Bhaktapur, Nepal; 2 Premier Dental Private Limited, Suryabinayak, Bhaktapur, Nepal; 3 Department of Periodontics and Oral Implantology, Kathmandu Medical College and Teaching Hospital, Duwakot, Bhaktapur, Nepal; 4 Department of Paedodontics and Preventive Dentistry, Kathmandu Medical College and Teaching Hospital, Duwakot, Bhaktapur, Nepal; 5 Department of Oral Medicine and Radiology, Kathmandu Medical College and Teaching Hospital, Duwakot, Bhaktapur, Nepal

**Keywords:** *gender*, *mandible*, *mental foramen*

## Abstract

**Introduction:**

Among many anatomical landmarks in the human skull, the mental foramen is a stable landmark on the mandible. The diverse morphology of the mandible indicates the specific characteristics of such anatomical structures in each individual. The aim of this study was to find out the mean distance of mental foramen from the inferior border of the mandible visiting the Outpatient Dental Department in a tertiary care centre.

**Methods:**

A descriptive cross-sectional study was conducted among patients undergoing orthopantomogram in the Outpatient Dental Department of a tertiary care centre from 3 February 2022 to 31 July 2022. Ethical approval was obtained from the Institutional Review Committee. The patients with complete dentition in the region of measurements were included in the study. A convenience sampling method was used. The point estimate was calculated at a 95% Confidence Interval.

**Results:**

Among 207 patients, the mean distance from the inferior border of the mandible to the lower border of the mental foramen was 11.83±1.83 mm (11.58-12.07, 95% Confidence Interval).

**Conclusions:**

The mean distance of mental foramen from the inferior border of the mandible was found to be similar to other studies done in similar settings.

## INTRODUCTION

The mandible is the hardest facial bone pertaining to sexual dimorphism. Among many anatomical landmarks in the human skull, the mental foramen (MF) is a stable landmark.^1^ Anatomically, it lies near the apices of premolars and the opening of the foramen is directed outward, upward, and posteriorly. MF is a very important landmark for the deposition of local anaesthesia and during various dental-related surgeries. During infancy, MF is located relatively closer to the inferior border of the mandible near the apices of the first molar tooth bud. In permanent dentition, it moves forward and lies just below the second premolar.^2^

Orthopantomogram (OPG) is a reliable and easily available radiographic tool in dentistry. Localisation of MF using OPG can help to identify an individual's age and gender.^3^ Only a handful of literature is evident on the radiographic localisation of MF in Nepal.

The aim of this study was to find out the mean distance of mental foramen from the inferior border of the mandible visiting the Outpatient Dental Department in a tertiary care centre.

## METHODS

This descriptive cross-sectional study was conducted among the patients visiting the Outpatient Department of Oral Pathology and Microbiology of Kathmandu Medical College and Teaching Hospital, Duwakot, Bhaktapur, Nepal. Data was collected from 3 February 2022 to 31 July 2022. Ethical approval was taken from the Institutional Review Committee (Reference number: 2401202202). Patients were informed about the procedure and written informed consent was obtained. The patients with complete dentition in the region of measurements were included in the study. The radiographic images of the mental foramen and the borders of the mandible that were not distinctly visible, images containing artefacts, and alveolar crest resorption in the premolar and first molar regions were excluded from the study. A convenience sampling method was used. The sample size was calculated using the following formula:


$$\begin{array}{ll} \text{n} & ={\text{Z}}^{2}\times \frac{\sigma^{\text{2}}}{{\text{e}}^{\text{2}}}\\ & =1.96^{2}\times \frac{1.69^{2}}{0.25^{2}}\\ & =176 \end{array}$$

Where,

n = minimum required sample size
Z = 1.96 at 95% Confidence interval (CI)
p = standard deviation taken from published literature, 1.69^4^
q = 1-p
e = margin of error

Hence, the minimum calculated sample size was 176. However, the final sample size taken was 207.

According to the criteria given by Yosue and Brooks appearance of MF was identified and type I MF (continuous) were included in the study.^5^ All the radiographs were taken using the Planmeca Proline 2006 EC machine with tube potential 60-80 KV, tube current 6-8 mA, total filtration 2.5 mm Al, focal spot 0.3 and time 18 s; using standard protocols. The radiographs were taken by a single radiographer. The collected radiographs were evaluated by Planmeca Romexis version 3.0.1 viewing software. The measurements were taken using the in-built tools of the software. The MF was located and marked on the image. The inferior border of the mandible was marked. The tangent from inferior border of the mandible was drawn to the lower border of the mental foramen on both the left and right sides of the arc All the measurements were taken in millimetres (mm). Three different readings were taken and their mean was used for further analysis to minimize intraobserver biases.

Data were entered using Microsoft Excel 2007 and analysed using IBM SPSS Statistics version 20.0. The point estimate was calculated at a 95% CI.

## RESULTS

Among 207 patients, the mean distance from the inferior border of the mandible to the lower border of the mental foramen was 11.83±1.83 mm (11.5812.07, 95% CI). A total of 105 (50.72%) were males and 102 (49.28%) were females. The total mean distance irrespective of arch side in males was 12.27±1.70 mm and for females 11.38±1.86 mm. The distance was 12.461±2.08 mm in males and 11.45±1.99 mm in females on the left side. Similarly, on the right side, the distance was 12.078±1.83 mm in males and 11.320±2.10 mm in females (Table 1).

**Table 1:** Distance of the lower border of mental foramen from the inferior border of the mandible (n= 207).

Mandibular arch side	Gender	Mean±SD (mm)
Left	Female	11.45±1.99
	Male	12.46±2.08
	Total	11.96±2.10
Right	Female	11.32±2.10
	Male	12.08 ±1.83
	Total	11.71±2.00

## DISCUSSION

Morphological variations of the skeletal structures are expected depending upon the population, which emphasised the need of population-specific anatomical standards.^6^ The mandible is the strongest bone which can withstand extreme conditions and persists well preserved for a longer time. The stability of MF remains throughout life. Resorption of the alveolar process occurs above the MF accounting relatively constant distance from it to the lower border of mandible throughout the life.^7,8^ Due to the stability of the basal bone and MF, these points were considered reference points for the present study.

In the present study, the mean distance from the inferior border of the mandible to the lower border of the mental foramen on both sides was noteworthy in males (right= 12.08±1.83 mm, left= 12.46±2.08 mm) compared to females (right= 11.32±2.10 mm, left= 11.45±1.99 mm). Our findings were similar to other studies conducted in different parts of the world, where mean was found to be for male (right= 12.67±2.61 mm, left= 12.58±2.49 mm), female (right= 11.46±2.92 mm, left= 11.25±3.18 mm), male (right= 11.79±0.97 mm, left= 11.89±2.40 mm), female (right= 11.41±0.65 mm, left= 11.40±0.66 mm), male (right= 16.13±2.31 mm, left= 15.88±1.92 mm), (right= 12.89±0.61 mm, left= 13.1±0.65 mm) respectively.^9-11^

Present study concluded that there is a difference in the mean value of LBMF to IBM in males and females on both sides of the mandible. The logical explanation for the difference in these measurements can be explained on the basis of the fact that sexual hormones such as androgens and estrogen contribute to the development of a morphologic difference in craniofacial skeletons between the genders.^12^ The finding of the present study showed, irrespective of arch side the total mean distance of IBM to LBMF in males was 12.27±1.70 mm and for females 11.38±1.86 mm, in accordance with studies where the mean values for males were 11.84±1.83 mm, 16.01±2.12 mm and for female, 11.4±0.64 mm, 12.9±0.64 mm respectively.^10,11^ As revealed from the study results, distance below the mental foramen (IBM to LBMF) on the left and right had similar values in both genders (left= 11.96±2.10 mm, right= 11.71±2.00 mm).

The findings of the present study suggest the effective localization of mental foramen for deposition of local anaesthesia in various surgical procedures as the total mean distance from IBM to LBMF was found to be 11.83±1.83 mm as in the previous study where the total mean was 10.27±1.69 mm.^13^ MF is considered a peremptory anatomical landmark from various surgical, aesthetic, and forensic odontology aspects. Unfortunately, sometimes MF is misdiagnosed as a radiolucent lesion apical to the second premolar. In such circumstances, knowledge of its positioning is most important for leading any intraoral procedure.^14,15^ The findings of the present study highlighted the importance of mental foramen and its implication as an adjunct tool for gender determination in forensic odontology as well as the area where the local anaesthesia can be deposited effectively. The application of 3D techniques such as Cone beam computed tomography (CBCT) along with the clinical correlations as well as statistical tests is recommended to validate the results. Further multicentric approach with a large sample size is advocated as the positioning of the MF is highly influenced by the projection of angulation.

The limitations of this study include a small sample size and a single-centre study. Furthermore, the localisation of mental foramen is highly influenced by the projection angle which may not have been uniform in all the cases in this study.

## CONCLUSIONS

The mean distance of mental foramen from the inferior border of the mandible was found to be similar to other studies done in similar settings.
